# Pharmacist’s recommendations of over-the-counter treatments for the common cold - analysis of prospective cases in Poland

**DOI:** 10.1186/s12875-021-01561-2

**Published:** 2021-10-30

**Authors:** Malgorzata Pietrusiewicz, Paulina Natalia Kopa-Stojak, Rafal Pawliczak

**Affiliations:** Department of Immunopathology, Faculty of Medicine, Medical University of Lodz, Lodz, Poland

**Keywords:** Common cold, Over-the-counter medication, Respiratory infections, Pharmacist, Common cold treatment

## Abstract

**Background:**

Common cold is a frequent illness in northern hemisphere between late autumn and early spring. Patients suffering from it frequently turn to pharmacists instead of physicians in order to receive medical advice and treatment. We studied its treatment advised by pharmacists in Poland, as well as evidence for the efficacy of their recommendations by utilizing a self-developed questionnaire and a study of existing literature.

**Methods:**

The data were collected by 27 pharmacists who worked in four large network community pharmacies in Lodz, Poland. The study took place from December 2019 to February 2020. Data were recorded only if the patient asked for pharmacy counselling for over-the counter (OTC) products due to common cold self-diagnosis and a product was sold. Pharmacists’ recommendations were compared with the results of a literature review of best evidence to determine appropriateness of the pharmacists’ decisions.

**Results:**

In four out of five cases the pharmacists recommended products contained paracetamol. In addition, in one out of three patient encounters they advised nasal decongestant, inosines and/or OTC mucolytics. There was a significant relationship between fever and recommendation frequency of some analgesics, inosines, mucolytics and sore throat products (OR > 1, *p* < 0.05); rhinorrhea and recommendation frequency of paracetamol, inosines, anti-histamines and alpha-mimetics (OR > 1, *p* < 0.05); cough and recommendation frequency of paracetamol, inosines, mucolytics and sore throat products (OR > 1, *p* < 0.05); and fatigue and recommendation frequency of paracetamol, acetylsalicylic acid, inosines and sore throat products (OR > 1, *p* < 0.05). The pharmacist recommendations were based on patients’ symptoms, product price, pharmaceutical company promotion and the financial incentive. In many cases their recommendations were not in line with current best practice recommendations.

**Conclusions:**

Our study suggests that the most common rationale for pharmacist recommendation on anti-common cold treatment was to take a “shotgun” approach. Pharmacists commonly made recommendations for products that lack strong evidence for efficacy (i.e. anti-viral agents) and are potentially unnecessary, based on presentation of the symptom. Reasons for this situation include lack of training, lack of time to evaluate the patient, lack of awareness of evidence as well as drug company marketing and financial incentives (i.e. fulfilling sale plans and target sale bonuses).

**Trial registration:**

The study was a non-interventional, observational research trial. The study registration was not required.

**Supplementary Information:**

The online version contains supplementary material available at 10.1186/s12875-021-01561-2.

## Background

Common cold is a quite frequent illness in northern hemisphere in late autumn, winter and early spring months. Usually it is caused by rhinoviruses, coronaviruses and adenoviruses, as a single or combined upper airway infection. To most patients the infection is self-limiting and lasts approximately 10–12 days [1]. Interestingly, there are only a few national guidelines on common cold treatment [2–4]. In most countries patients suffering from this disease turn to pharmacists instead of physicians in order to receive medical advice and treatment. In one study 68% of patients consulted pharmacists and only 28% asked physicians for advice [5]. Therefore, the pharmacists’ role in common cold treatment is crucial. The pharmacists’ qualifications in counseling the patients with simple diseases differ internationally and are related to local law and pharmacist training. In some countries it focuses on advising treatment and is formalized as “pharmacist counselling”. In Poland, the pharmacists’ role is limited to selling prescribed and over-the-counter medications [6, 7]. The pharmacy profession describes their role in dispensing over-the counter (OTC) products as “selecting the best medication according to reported symptoms, while taking into account the safety and efficiency of the product”. In addition, pharmacists should inform the patient about the product characteristic, dosage, possible side effects and drug interactions. If necessary, they should also remind the patients to consult physician [8–10]. The results of one large study suggested that 56% of responders trusted their pharmacists as drug consultants [11]. In some countries 73.8% of pharmacist counseling is related to common cold [12]. The number of OTC medications which might be used in common cold treatment is enormous and growing. It can be difficult and unsafe for an average patient to select the most appropriate ones. Most of them are soluble compositions of several drugs with different mechanisms of action, focusing on relieving selected common cold symptoms. In most cases they are combinations of nonsteroidal anti-inflammatory drugs (NSAIDs), anti-histamines (usually old first generation), mucolytics, cough suppressants (central action only), and alpha1-mimetics [13–17]. To ensure fast onset of action, in most combinations soluble forms in powder are available. Moreover, several preparations with ascorbic acid combined with one or two aforementioned drugs are also available. Additionally, preparations with honey and herbal products are also used in common cold treatment [18–21]. Some of them have proven efficacy and safety, some not [18, 22–31]. All of them are available without prescription.

Therefore, it is of interest to analyze how patients choose OTC medications to treat common cold. There is no data available to show which medications are advised by pharmacists. Moreover, the motivation of pharmacists to advise specific medications is unknown.

## Methods

### Study characteristic

The study was performed in accordance with Article 2(c) of The Directive 2001/20/EC of the European Parliament and the Council as non-interventional, observational trials. The original, self-developed questionnaire was applied in four large network community pharmacies in Lodz, Poland, to all the patients who came for advice. Before starting this observational study, each pharmacist received guidelines (presented by a member of the research team) on how these consultations should be conducted. In addition, they also received recommendations on what questions should be raised during the consultation and how to fill in the data collection form in order to collect only the data valid for further analysis.

### Patients

Only adult patients (aged 18 years and over) were included in the study. The data were collected only if the patient asked for pharmacy counselling due to common cold self-diagnosis (based on the most common symptoms). If the patient was suffering from other symptoms or/and had a prescription for other medications, the data collection form was not filled and the data were not recorded. The following symptoms were recorded: cough, rhinitis, headache, fatigue or fever not exceeding 37.7 °C.

### Collected data

The data collection form was filled by a pharmacist after a pharmacy counselling session resulting in dispensing an OTC medication to be utilized in common cold treatment. The data collection forms were collected from December 2019 to February 2020. They included data on age and sex of the pharmacy patient, number and type of common cold symptoms, type of products recommended by the pharmacist after each patient encounter and a rationale of the pharmacist’s recommendation on the specific anti-common cold treatment in each individual case. The anti-common cold treatment recommendation section was divided into three sections (based on product formulation): soluble products (i.e. soluble powders in sachets, soluble tablets) including paracetamol, acetylsalicylic acid and magnesium metamizole; tablets/capsules including paracetamol, acetylsalicylic acid, ibuprofen, pseudoephedrine, anti-viral agents, herbal and homeopathic products; and add-on treatment, such as alpha-mimetics (nasal spray and gels), mucolytics (syrups), anti-histamines and products for sore throat. English translation of the data collection form is presented as a supplementary material (see Supplementary file 1).

### Searching strategy and study selection

To determine if the pharmacists’ recommendations were consistent with best evidence or expert recommendation from clinical practice guidelines, we identified the existing evidence on these OTC products from a search of the Cochrane Library. The search was performed in May and June 2021, based on the terms: “common cold”, “adult”, “effective treatment”. We focused on publications in English or Polish. For this analysis we chose systematic reviews and randomized clinical trials on the effectiveness of common cold treatment (for adults). Additional publications summarizing randomized controlled trials on treatment strategies were chosen from PubMed database (if Cochrane Library does not provide access to findings or only protocol was available). Supplementary file 2 summarizes the searching strategy. From each individual publication we extracted data on type of study, number of enrolled patients, treatment strategy and treatment effectiveness. Based on the data we prepared a summary of anti-common cold treatment options with beneficial effect, with potential/unclear effect or with no evidence of effect (see Supplementary Table 1). Finally, we compared the results obtained in our observational study with those from summarized clinical research on treatment effectiveness, then discussed the appropriateness of pharmacist recommendations of specific classes of anti-common cold products.

### Statistical analysis

Statistical analysis was performed using Statistica 13.3 (StatSoft Inc., Tulsa, OK). The proportion of patients receiving each treatment is reported as a fraction of number of cases to all cases, and as percentage of patient encounters with anti-common cold medication recommendation. The Pearson’s Chi square test was used to compare the frequency of receiving particular medications for patients reporting different symptoms. Odds ratio (with 95% CI) was calculated to determine the relationship between presence of specific common cold symptoms and frequency of medication recommendation. The data with *p*-value < 0.05 were considered statistically significant.

## Results

### Characteristics of the patients

Five hundred and two patient cases were examined. The majority of patients (53.1%) were women. Almost 2/3 of patients were between 18 and 40 years old (64.7%). Table 1 summarizes the demographical data.

**Table 1:** Patients characteristics

Sex [n;%]	
Female	264 (53.1%)
Male	233 (46.9%)
Age [n;%]	
18–40 years	325 (64.7%)
41–60 years	145 (28.9%)
61–90 years	32 (6.4%)

**Fig. 1:**
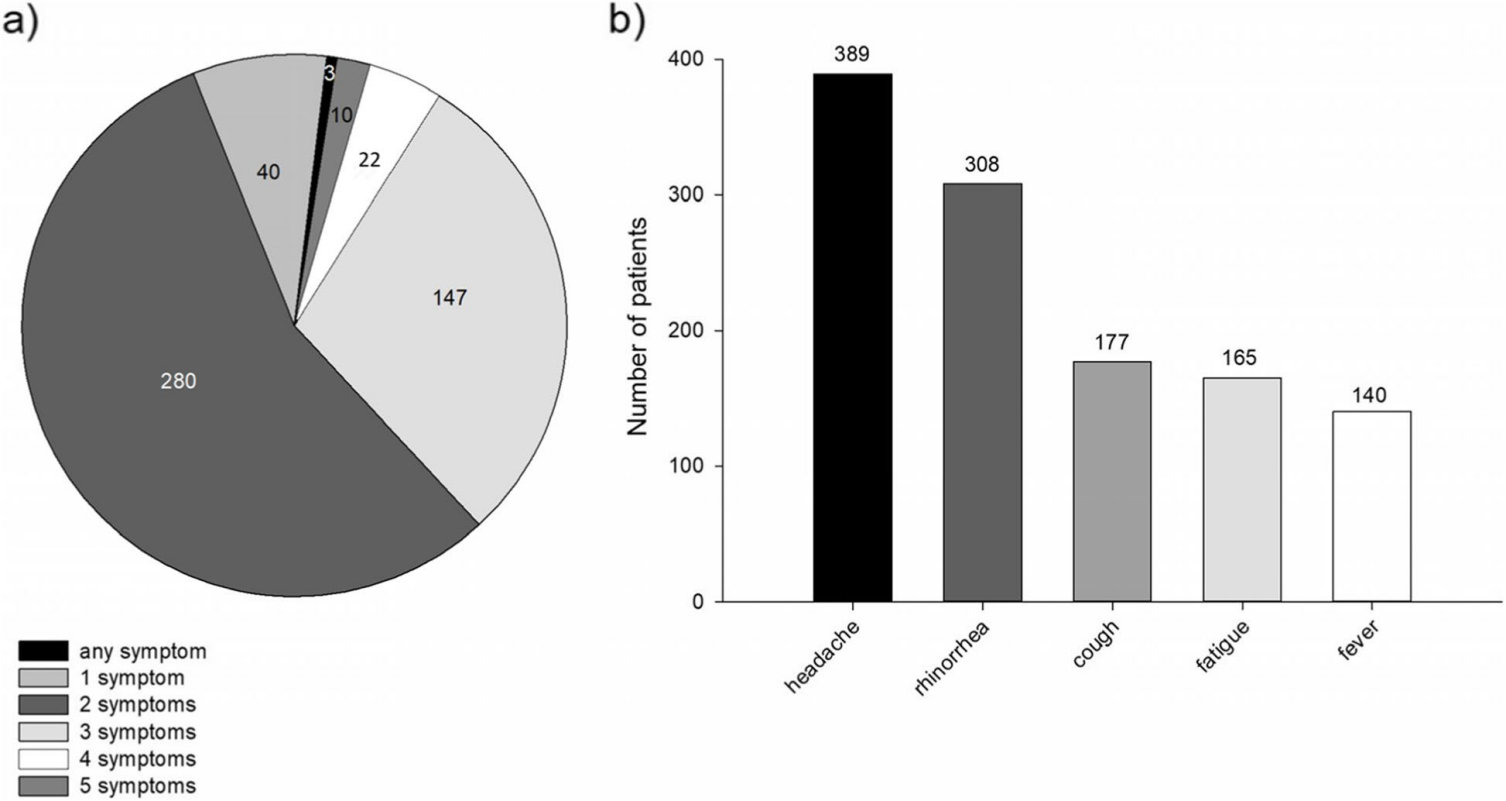
Common cold symptoms reported by the patients. Common cold symptoms reported in 502 patient encounters during pharmacists’ recommendations of OTC products (**a**). Frequency of common cold symptoms reported by patients (**b**). The results are presented as raw numbers of patient encounters with anti-common cold medication recommendation

The majority of patients (280/502 (55.8%)) reported two out of 5 symptoms listed in the study questionnaire. Interestingly, 3/502 (0.6%) patients did not identify any symptoms from the list and reported their self-diagnosis as “common cold”, and 10/502 (2%) patients identified all 5 symptoms from the list (Fig. 1a).

The most frequently reported symptom was headache, present in 389/502 (77.5%) cases, followed by rhinorrhea 308/502 (61.4%) cases, and cough 177/502 (35.1%). Fatigue and elevated body temperature were present in 165/502 (32.9%) and 140/502 (27.9%) patients, respectively (Fig. 1b).

### Class of anti-common cold treatment mostly recommended by pharmacists

**Fig. 2:**
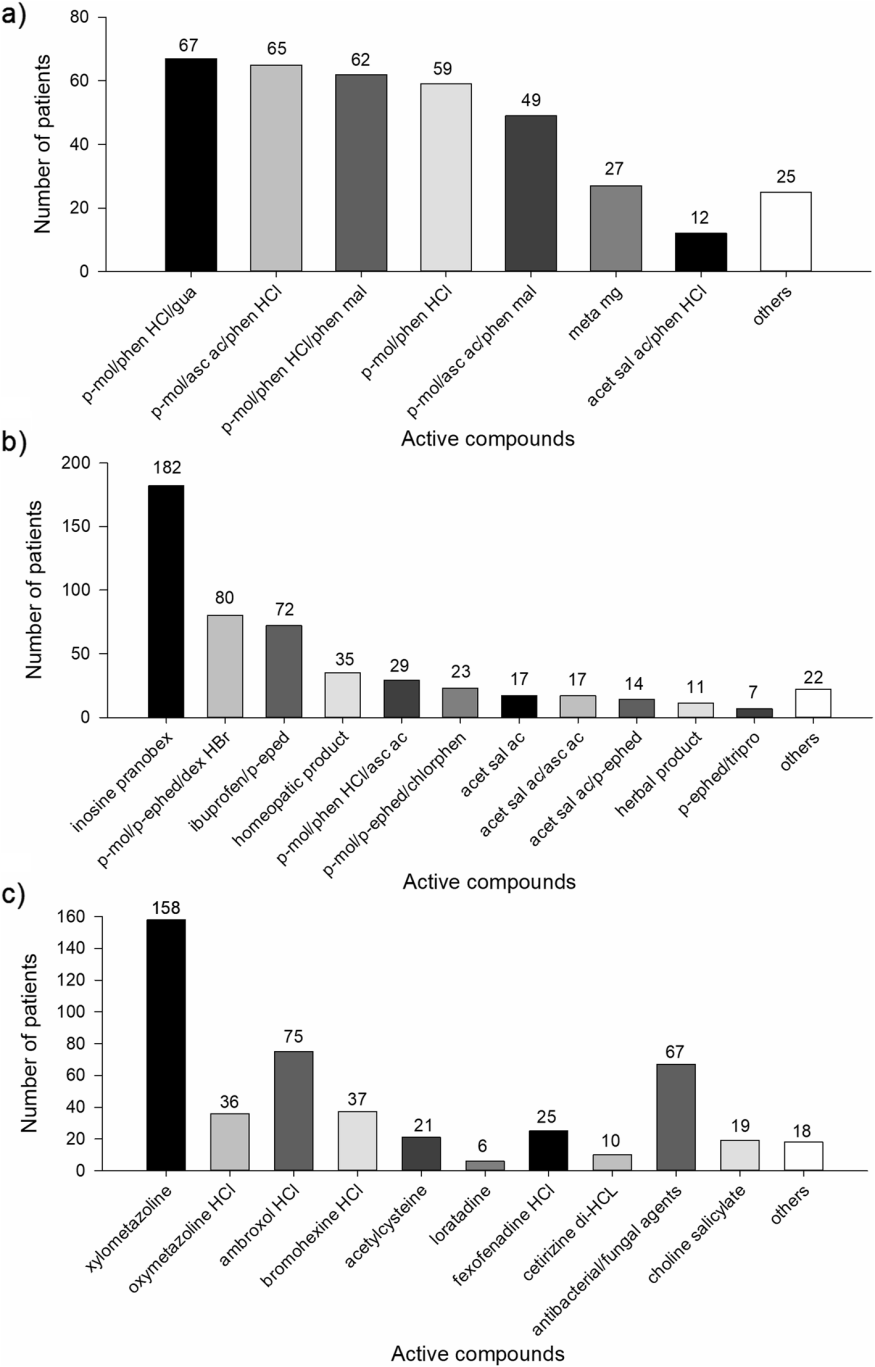
Anti-common cold treatment recommendations. Frequency of recommending soluble formulations (**a**), tablets/capsules (**b**), or add-on (**c**) OTC anti-common cold products by the pharmacists in 502 patient encounters. The results are presented as raw numbers of patient encounters with anti-common cold medication recommendation. Abbreviations: *acet sal ac* – acetylsalicylic acid; *asc ac* – ascorbic acid; *chlorphen* – chlorpheniramine; *dex HBr* – dextromethorphan hydrobromide; *gua* – guaifenesin; *meta mg* – metamizole magnesium; *p-mol* – paracetamol; *phen HCl* – phenylephrine HCl; *phen mal* - pheniramine maleate; *p-ephed* – pseudoephedrine; *tripro* – triprolidine

Soluble form of medication was recommended in 339/502 (67.5%) cases, where the most frequent were a combination of paracetamol, guaifenesin and phenylephrine HCL (utilized in 67/502 (13.3%) patient encounters), paracetamol, ascorbic acid and phenylephrine HCL (advised in 65/502 (12.9%) patient encounters), or products composed of paracetamol, phenylephrine HCL and pheniramine maleate (recommended in 62/502 (12.4%) patient encounters), and a simple combination of paracetamol and phenylephrine HCL (advised in 59/502 (11.8%) patient encounters) (Fig. 2a).

Oral tablets/capsule medications were advised in 404/502 (80.5%) cases, where the most frequent were inosine pranobex (recommended in 182/502 (36.3%) patient encounters), and a combination of paracetamol, pseudoephedrine and dextromethorphan hydrobromide (advised in 80/502 (15.9%) cases). Tablets containing a combination of ibuprofen and pseudoephedrine were recommended in 72/502 (14.3%) cases (Fig. 2b).

Add-on treatment was recommended in 352/502 (69.7%) patient encounters, mostly included: nasal spray containing alpha-mimetics (i.e. xylometazoline, oxymetazoline HCL) utilized in 194/502 (38.6%) cases, mucolytics (such as acetylcysteine, ambroxol HCL and bromohexine HCL) advised in 133/502 (26.5%) cases or antibacterial/antifungal agents for sore throat, recommended in 67/502 (13.3%) patient encounters (Fig. 2c).

The pharmacists’ recommendations for anti-common cold treatment varied between one to seven different medications. The most frequently advised combinations consisted of three or two different products, in 184/502 (36.7%) and 150/502 (29.9%) patient encounters, respectively. A single anti-common cold medication was recommended in 77/502 (15.3%) cases. In 34/502 (6.8%) cases 4 products, in 28/502 (5.6%) cases 5 products, in 25/502 (5%) cases 6 products and in 4/502 (0.7%) cases 7 different medications were recommended (Table 2).

**Table 2:** Quantity of anti-common cold medications recommended by the pharmacists

Number of medications ^a^	Quantity of recommendations [n;%] ^b^	Combinations of anti-common cold treatment [n;%]^c^	
1	77 (15.3%)	1 soluble	32 (6.4%)
		1 tablets	43 (8.6%)
		1 add-on	2 (0.4%)
2	184 (36.7%)	2 tablets	28 (5.6%)
		2 add-on	1 (0.1%)
		1 soluble/1 add-on	58 (11.6%)
		1 soluble/1 tablets	44 (8.8%)
		1 tablets/1 add-on	53 (10.6%)
3	150 (29.9%)	3 add-on	1 (0.1%)
		1 soluble/2 add-on	6 (1.2%)
		1 soluble/2 tablets	1 (0.1%)
		1 tablets/2 add-on	5 (1%)
		1 add-on/2 tablets	25 (5%)
		1 soluble/1 tablets/1 add-on	112 (22.3%)
4	34 (6.8%)	1 tablets/3 add-on	1 (0.1%)
		2 tablets/2 add-on	4 (0.7%)
		1 soluble/1 tablets/2 add-on	24 (4.8%)
		1 soluble/2 tablets/1 add-on	4 (0.7%)
		2 soluble/1 tablets/1 add-on	1 (0.1%)
5	28 (5.6%)	1 soluble/2 tablets/2 add-on	14 (2.8%)
		2 soluble/1 tablets/2 add-on	3 (0.6%)
		2 soluble/2 tablets/1 add-on	1 (0.1%)
		1 soluble/1 tablets/3 add-on	10 (2%)
6	25 (5%)	1 soluble/1 tablets/4 add-on	1 (0.1%)
		1 soluble/2 tablets/3 add-on	6 (1.2%)
		2 soluble/1 tablets/3 add-on	1 (0.1%)
		2 soluble/2 tablets/2 add-on	17 (3.4%)
7	4 (0.7%)	2 soluble/2 tablets/3 add-on	2 (0.4%)
		2 soluble/3 tablets/2 add-on	2 (0.4%)

^a^ Number of recommended anti-common cold medications (presented as a raw number)

^b^ Number of patient encounters where a given number of medications were recommended, presented as raw numbers and, in brackets, as percentage of total 502 patient encounters

^c^ Anti-common cold treatment was divided into three sections: soluble products including paracetamol, acetylsalicylic acid and magnesium metamizole; tablets/capsules including paracetamol, acetylsalicylic acid, ibuprofen, pseudoephedrine, anti-viral agents, herbal and homeopathic products; and add-on treatment, such as alpha-mimetics, mucolytics, anti-histamines and products for sore throat. Data presented as raw numbers of the patient encounters with anti-common cold medication recommendation and as percentage of the pharmacists’ recommendations in 502 patient encounters

The most frequent pharmacist recommendation was a combination of 3 different products - one soluble, one tablet/capsule formulation and one add-on common cold medication, reported in 112/502 (22.3%) patient encounters. Here, the most frequent combination comprised paracetamol with anti-viral product and with mucolytics, recommended in 22/112 (19.6%) cases. The second most popular combination comprised paracetamol with anti-viral agents and alpha-mimetics, advised in 16/122 (14.3%) patient encounters. The third most frequent recombination (advised in 58/502 (11.6%) cases) comprised a soluble medication with add-on anti-common cold treatment (i.e. ibuprofen/pseudoephedrine with alpha-mimetics or paracetamol with alpha-mimetics), and the fourth most frequent recombination was a combination of one medication in tablet formulation with add-on treatment (advised in 53/502 patient encounters) (Table 2).

### Rationale of pharmacist recommendations for anti-common cold treatment

**Fig. 3:**
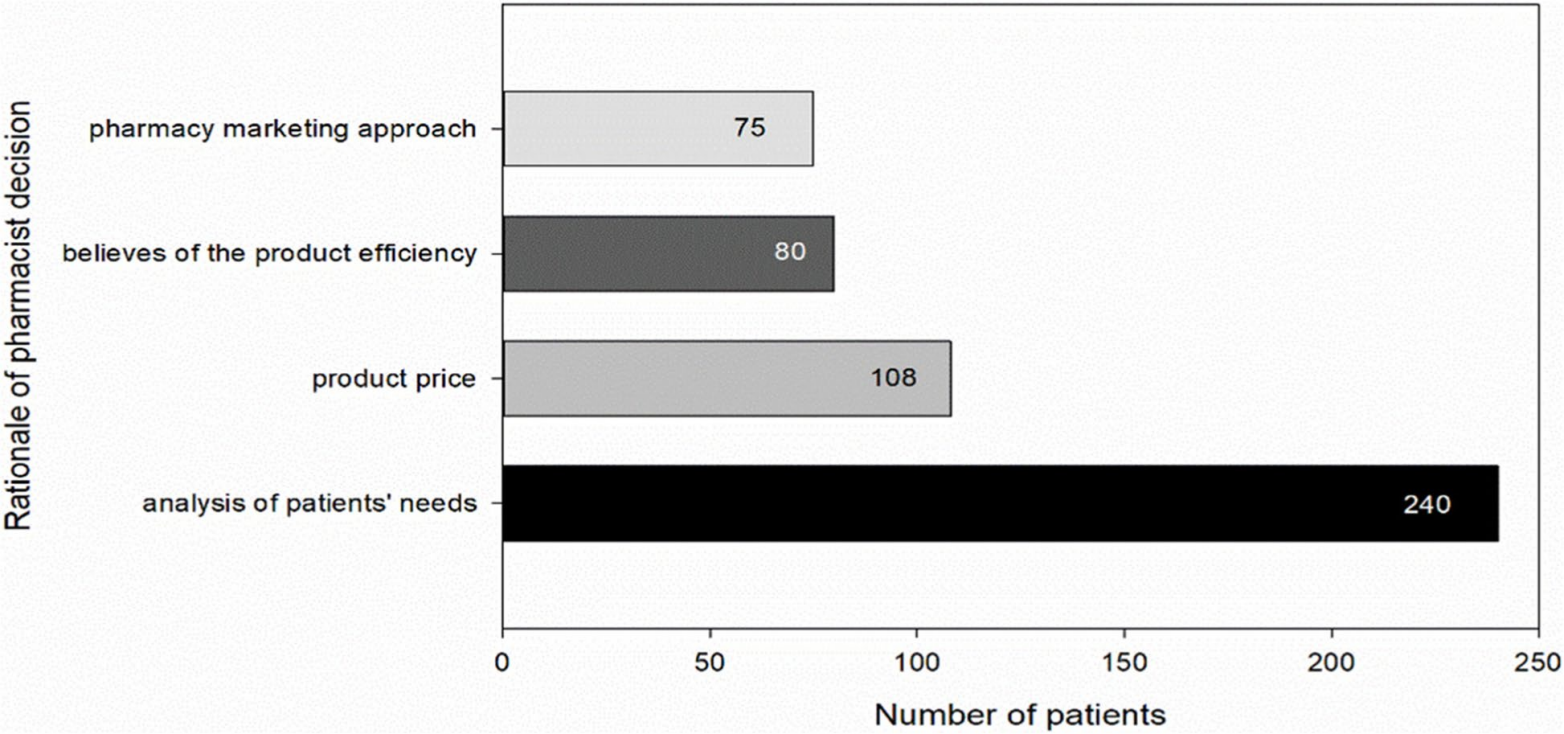
The rationale of the pharmacists’ decisions. Reasons for advising specific medications by the pharmacists in order to treat common cold based on OTC medication recommendations in 502 patient encounters. The results are presented as raw numbers of patient encounters with anti-common cold medication recommendations

The reasons behind advising specific medication by pharmacists in order to treat common cold are always unspecified. In our study, in 240/502 (47%) patient encounters the pharmacists declared that their approach to patient treatment was based solely on patients’ complaints, history and analysis of the patient’s needs. Moreover, in 108/502 (21.5%) patient encounters the pharmacists declared that the price of the drug was a major factor. In addition, in 80/502 (15.9%) cases the pharmacists advised a common cold medication basing on their belief in the efficacy of the drug in current clinical situation. In 75/502 (14.9%) patient encounters the pharmacists declared that a major reason for advising a specific medication was a marketing approach from inside the pharmacy or outside, for financial reward or other benefits paid by pharmaceutical industry or its representatives (Fig. 3).

### Anti-common cold treatment recommendations based on the selected clinical symptoms

#### Headache

It was of interest to analyze if specific common cold symptoms drive advice towards causative OTC treatment. Headache reported by patients did not deliver any specific treatment. More than two-thirds of patients with headache received a combination of medications containing NSAIDs. In 150/389 (38.6%) cases they received alpha-mimetics, and in 27/389 (6.9%) cases an old, first generation antihistamine (Table 3).

**Table 3:** Products advised by the pharmacists for a selected clinical symptoms of common cold

Symptom	Patients receiving medications [n]	Statistics
headache	paracetamol [329]	*p* > 0.05
	acetylsalicylic acid [41]	
	magnesium metamizole [15]	
	inosines [140]	
	homeopathics [17]	
	herbal products [5]	
	pseudoephedrine [67]	
	alpha-mimetics [150]	
	anti-histamines [27]	
	mucolytics [78]	
	sore throat products [53]	
fever	paracetamol [127]	for paracetamol with ascorbate acid and pheniramine maleate OR = 2.105, 95%CI 1.152–3.843; *p* = 0.022
	acetylsalicylic acid [25]	for acetylsalicylic acid OR = 3.901, 95%CI 1.445–10.463; *p* = 0.009; for acetylsalicylic acid with phenylephrine HCL and chlorphenamine OR = 3.758, 95%CI 1.172–12.045; *p* = 0.04
	magnesium metamizole [14]	for magnesium metamizole OR = 2.983, 95%CI 1.365–6.52; *p* = 0.008
	inosines [68]	for inosines OR = 4.754, 95%CI 1.832–12.34; *p* = 0.001
	homeopathics [14]	*p* > 0.05
	herbal products [8]	
	pseudoephedrine [17]	
	alpha-mimetics [54]	
	anti-histamines [7]	
	mucolytics [49]	for ambroxol HCL OR = 2.664, 95%CI 1.353–5.242; *p* = 0.006
	sore throat products [46]	for choline salicylate OR = 3.017, 95%CI 1.199–7.591, *p* = 0.028; for chlorchinaldol OR = 3.222, 95%CI 1.491–6.96, *p* = 0.004; for amylmetacresol with levomenthol and 2,4-dichlorobenzol OR = 3.677, 95%CI 1.515–8.933, *p* = 0.005
rhonorrhea	paracetamol [265]	for paracetamol with phenylephrine HCL OR = 8.036, 95%CI 3.154–20.477, *p* < 0.001
	acetylsalicylic acid [28]	*p* > 0.05
	magnesium metamizole [4]	
	inosines [90]	for inosines OR = 10.509, 95%CI 4.153–26.591, *p* < 0.001
	homeopathics [13]	*p* > 0.05
	herbal products [5]	
	pseudoephedrine [72]	
	alpha-mimetics [166]	for xylometazoline in nasal spray OR = 8.96, 95%CI 2.726–29.452, *p* < 0.001; for xylometazoline gel form OR = 4.366, 95%CI 2.508–7.603, *p* < 0.001
	anti-histamines [28]	for fexofenadine HCL OR = 4.987, 95%CI 1.446–16.95, *p* = 0.009
	mucolytics [52]	*p* > 0.05
	sore throat products [41]	
cough	paracetamol [185]	for paracetamol, ascorbic acid and phenylephrine HCL OR = 5.667, 95%CI 1.132–28.378, *p* = 0.046; for paracetamol, ascorbic acid and pheniramine maleate OR = 2.261, 95% CI 1.242–4.092, *p* = 0.01; for paracetamol, ascorbic acid and phenylephrine HCL OR = 4.515, 95%CI 1.821–11.196, *p* < 0.001; for paracetamol, phenylephrine HCL and ascorbic acid (in a form of tablets) OR = 2.771, 95%CI 1.292–5.945, *p* = 0.012
	acetylsalicylic acid [27]	*p* > 0.05
	magnesium metamizole [7]	
	inosines [81]	for inosines OR = 1.812, 95%CI1.175–2.794, *p* = 0.009
	homeopathics [15]	*p* > 0.05
	herbal products [3]	
	pseudoephedrine [14]	
	alpha-mimetics [53]	
	anti-histamines [14]	
	mucolytics [105]	for bromohexide HCL OR = 18.391, 95%CI 6.396–52.877, *p* < 0.001; of ambroxol HCL OR = 9.57, 95%CI 3.869–23.671, *p* < 0.001; of acetylcysteine OR = 8.527, 95%CI 2.822–25.759, *p* < 0.001
	sore throat products [47]	for chlorchinaldol OR = 4.222, 95%CI 1.867–9.546, *p* < 0.001
fatigue	paracetamol [169]	for paracetamol with ascorbic acid and phenylephrine HCL OR = 3.378, 95%CI 1.431–7.979, *p* = 0.007; for paracetamol with phenylephrine HCL and ascorbic acid (in form of tablets) OR = 2.307, 95%CI 1.086–4.902, *p* = 0.043; for paracetamol with pseudoephedrine and dextromethorphan OR = 1.917, 95%CI 1.131–3.252, *p* = 0.021
	acetylsalicylic acid [26]	for acetylsalicylic acid with pseudoephedrine OR = 3.831, 95%CI 1.263–11.62, *p* = 0.024; for acetylsalicylic acid with ascorbic acid OR = 3.041, 95% CI 1.136–8.141, *p* = 0.04
	magnesium metamizole [5]	*p* > 0.05
	inosines [73]	for inosines OR = 1.715, 95%CI 1.107–2.657, *p* = 0.021
	homeopathics [12]	*p* > 0.05
	herbal products [2]	
	pseudoephedrine [10]	
	alpha-mimetics [44]	
	anti-histamines [8]	
	mucolytics [50]	
	sore throat products [49]	for choline salicylate OR = 4.718, 95%CI 1.76–12.65, *p* = 0.002; for amylmetacresol with levomenthol and 2,4-dichlorobenzol OR = 3.517, 95%CI 1.428–8.664, *p* = 0.008

#### Fever

There was a statistical significance between fever complaints and utilization of various forms of NSAIDs (*r* = 0.69 *p* < 0.05). A soluble, fast-acting form was the most common, as compared to tablets (122/140 (87.5%) cases vs. 64/140 (45.7%) cases; *p* < 0.05, respectively). In 68/140 (48.6%) cases, the pharmacists recommended anti-viral and one-third of patients with fever took mucolytics (advised in 49/140 (35%) cases). In addition, there was a relationship between patient encounters with reported fever and recommendation frequency for acetylsalicylic acid, magnesium metamizole, paracetamol, inosines, mucolytics and sore throat products (OR > 1, *p* < 0.05) (Table 3).

#### Stuffy nose

Alpha-mimetics in nasal spray formulations were used in 166/308 (53.8%) patients, with rhinorrhea as the dominant symptom. The most common medication included a 0.1% xylometazoline solution in nasal spray (100/308 (32.4%) cases; *r* = 0.999; *p* < 0.05). The gel form of xylometazoline was the second most frequent nasal preparation, used in 38/308 (12.3%) patients (*r* = 0.993; *p* < 0.05). In addition, in 54/308 cases the pharmacists recommended a combination of paracetamol with phenylephrine HCL, and inosines were advised in 90/308 (29.2%) cases. There was a relationship between patient encounters with reported stuffy nose and recommendation frequency of xylometazoline, paracetamol, anti-histamines and inosines (OR > 1, *p* < 0.05) (Table 3).

#### Cough

Cough is one of the most widespread symptoms of common cold. Almost two-thirds of patients with cough received medications with soluble paracetamol and mucolytics (123/177 (69.4%) cases and 105/177 (59.3%) cases, respectively). In addition, almost half of them received products containing paracetamol (62/177 (35%) cases) or acetylsalicylic acid (21/177 (11.9%) cases) in the form of tablets/capsules. In addition, over one-third of patients received anti-viral products, alpha-mimetics or medications for sore throat (81/177 (45.7%) cases, 53/177 (29.9%) cases and 47/177 (26.5%) cases, respectively). There was a significant relationship between cases with reported cough and recommendation frequency for products containing inosines, mucolytics and chlorchinaldol (OR > 1, *p* < 0.05) (Table 3).

#### Fatigue

Patients who reported fatigue as the main common cold symptom received products containing soluble paracetamol (107/165 (64.8%) cases) and anti-viral products (in 73/165 (44.3%) cases). Almost one-third of patients with fatigue received alpha mimetics (recommended in 44/165 (26.6%) cases), mucolytics (recommended in 50/165 (30.4%) cases) and medications for sore throat (recommended in 49/165 (29.8%) cases). Interestingly, there was a relationship between cases with reported fatigue and recommendation frequency of acetylsalicylic acid, paracetamol, inosines and products for sore throat (OR > 1, *p* < 0.05) (Table 3). Patients with common cold frequently suffer from only a limited number of main symptoms, which might drive them to express a precise need for medication. To assess the pharmacist approach to these needs we analyzed the treatment approach in the most common combinations of symptoms.

### Anti-common cold treatment recommendations of the most common symptom combinations

Paracetamol in soluble formulation was most frequently received by the patients suffering from: headache and fever (in 11/17 (64.7%) patients), fever and cough (in 7/13 (53.8%) patients), fever and rhinitis (in 11/12 (91.7%) patients), fever and fatigue (in 6/7 (85.7%) patients), cough and rhinitis (in 8/9 (88.9%) patients), or cough and fatigue (in 10/13 (76.9%) patients). Patients complaining about headache and rhinorrhea mostly received rhinorrhea medications (in 92/147 (62.3%) patients). Among patients with headache and cough as a main symptoms, the most frequent recommendations were cough medications (in 21/26 (80.8%) patients), and for these with headache and fatigue - inosines (in 16/34 (47.1%) patients). All patients with rhinitis and fatigue received paracetamol in soluble formulation and rhinorrhea medications (Table 4).

**Table 4:** Treatment of the most common cold symptom combinations

Symptoms [n]	Patients receiving medications [n;%]								
	paracetamol (soluble)	paracetamol (tablets)	acetylsalicylic acid	inosines	ibuprofen	herbal and homeopathic medications	rhinorrhea medication^**a**^	cough medications	sore throat medications
headache rhinorrhea (147)	79 (53.7%)	24 (16.3%)	6 (4.1%)	29 (19.7%)	45 (30.6%)	4 (2.7%)	**92 (62.3%)**	2 (1.4%)	2 (1.4%)
headache fatigue (34)	14 (41.2%)	11 (32.4%)	9 (26.5%)	**16 (47.1%)**	0 (0%)	0 (0%)	2 (5.9%)	3 (8.8%)	6 (17.6%)
headache cough (26)	15 (57.7%)	11 (42.3%)	4 (15.4%)	11 (42.3%)	0 (0%)	3 (11.5%)	6 (23.1%)	**21 (80.8%)**	2 (7.7%)
headache fever (17)	**11 (64.7%)**	4 (23.5%)	3 (17.6 5)	9 (52.9%)	0 (0%)	4 (23.5%)	4 (23.5%)	5 (29.4%)	7 (41.2%)
cough fatigue (13)	**10 (76.9%)**	5 (38.5%)	1 (7.7%)	5 (38.5%)	0 (0%)	1 (7.7%)	3 (23.1%)	9 (69.2%)	3 (23.1%)
fever cough (13)	**7 (53.8%)**	6 (46.2%)	3 (23.1%)	5 (38.5%)	1 (7.7%)	3 (23.1%)	5 (38.5%)	4 (30.8%)	6 (46.2%)
fever rhinorrhea (12)	**11 (91.7%)**	5 (41.7%)	3 (25%)	7 (58.3%)	1 (8.3%)	4 (33.3%)	10 (83.3%)	10 (83.3%)	4 (25%)
rhinorrhea cough (9)	**8 (88.9%)**	0 (0%)	2 (22.2%)	5 (55.6%)	1 (11.1%)	1 (11.1%)	4 (44.4%)	7 (77.8%)	1 (11.1%)
fever fatigue (7)	**6 (85.7%)**	1 (14.3%)	3 (42.9%)	1 (14.3%)	1 (14.3%)	1 (14.3%)	1 (14.3%)	2 (28.6%)	1 (14.3%)
rhinorrhea fatigue (2)	**2 (100%)**	0 (0%)	1 (50%)	0 (0%)	1 (50%)	0 (0%)	**2 (100%)**	0 (0%)	0 (0%)

The mostly recommended anti-common cold medications for given symptoms are marked in bold

^a^ Alpha-mimetics and anti-histamines

### Discussion

To our best knowledge, this is the first study assessing pharmacist counselling focused on class, types and formulas of medication advised for common cold treatment in Poland. A similar study to ours was published on counselling efficacy and communication, where the counselling was found to be suboptimal. However, in *Mináriková* et al. the study mainly focused on the counselling process, rather than the appropriateness of product recommendations [6]. Every adult experiences 2–3 episodes of cold annually. In children this frequency is much higher, up to 5–6 episodes per year [32, 33].

### Comparison with existing literature on best practice

A vast majority of pharmacists in our study (almost 9 out of 10 patient encounters) recommended medications containing a combination of paracetamol with additional active compounds against common cold symptoms. The amount of paracetamol recommendations exceeded these for NSAIDs (advised in over one quarter of cases). Analysis of clinical trials shows that analgesics effectively work against headache, muscle pain, sneezing, but show no significant improvement for cough score, reduction of cold duration or total symptom score. Paracetamol has proven short-term effectiveness against nasal obstruction and relieving headache, but lacks significant effect on sore throat or cough [34]. Ibuprofen acts more effectively than paracetamol against sneezing and fever, but there is no clear evidence for improving respiratory symptoms [35]. As clinical data show, nasal congestion may also be effectively reduced by using decongestants, which were also commonly advised by the pharmacists in our study (in one third patient encounters). Clinical trials show small clinical effect for multi-dose of nasal decongestant in common cold treatment [36]. The use of xylometazoline in monotherapy shows clinically relevant decongestant effect [37], and intranasal application of ipratropium bromide acts against rhinorrhea, but without effectiveness against nasal congestion [38]. Combined treatment with xylometazoline and ipratropium bromide reduces both nasal congestion and rhinorrhea [37]. Furthermore, clinical trials indicate that antihistamine monotherapy has no clinical effect (especially for mid- and long-term usage) [39], but its combination with analgesics and/or decongestant show some beneficial effects in adults [16]. Also, in our study we observed that pharmacists recommended second generation anti-histamines (in 1 out of 5 cases) in a combined therapy for patients with common cold symptoms.

Analysis of clinical trial results indicate that nasal saline irrigation shows possible but unclear benefits in relieving symptoms of acute upper respiratory tract infection, but due to limited number of evidence of its effectiveness further large scale randomized clinical trials are needed [40]. Current evidence suggest that using garlic, zinc and vitamin C possess rather preventive than curative effect against common cold [26–28]. A variety of tested products, statistical heterogeneity of the results between different trials and questionable clinical relevance did not allow to support recommending echinacea [29], Chinese medical herbal products [30] or *Pelargonium sidoides* extract [31] for common cold treatment. The results of our study indicate that despite unclear clinical relevance of herbal or homeopathic products, they were recommended by the pharmacists in 1 out of 10 cases.

There is no clinical relevance to using antibiotics [41], applying intranasal corticosteroids [42], or antihistamine monotherapy for reducing separate common cold symptoms [39]. Finally, there is no clinically relevant benefit of using antivirals in upper respiratory tract infection. In addition, currently no antiviral agent is licensed to effective use in common cold treatment [43]. Interestingly, in one-third of patient encounters the pharmacists recommended anti-viral products. Moreover, one out of three patients received mucolytics, instead of cough suppressants for cough treatment. Supplementary file 3 contains a summary of the research on the effectiveness of symptomatic common cold treatment options in adults. Taken together, this indicates that pharmacist recommendations are not always in line with current best practice recommendation. This particularly applies to recommending anti-viral products, as they are ineffective in upper respiratory tract infection, or mucolytic agents, which are usually advised for lower respiratory tract infection.

Most clinical practice guidelines of common cold treatment are country and organization specific. The most popular are the United States Center For Disease Control guidelines, which recommend treating this disease with a combination of the following medications: 1) pain relievers (non-steroidal anti-inflammatory drugs) 2) nasal decongestants (alpha-mimetics) 3) cough suppressants 4) anti-histamines 5) expectorants [44]. *van Driel* found similar results in their clinical review [45]. Thus, the treatment is mostly based on symptoms presented by the patient, and curative treatment is not possible [46, 47].

Presented results clearly indicate that in four out of five cases the pharmacists recommended products containing paracetamol (where two out of three were in soluble form). The most frequent choice was a combination of paracetamol, guaifenesin and phenylephrine, which seems to, at least in part, treat the most common symptoms, such as headache, rhinitis, fever and cough. While paracetamol works mainly by its anti-pain action, guaifenesin is an old expectorant agent with numerous side effects and unproven effect on cough frequency, although there is one review suggesting otherwise [48]. Phenylephrine might be effective in nasal congestion, but may also have some important adverse events [49, 50]. The second most frequent combination was paracetamol with phenylephrine and pheniramine. In this case, the old, first generation antihistamine drug was used instead of alpha-mimetic. Pheniramine may cause several adverse events and should not be used when drugs with better safety records are available [51]. Another frequently advised combination was paracetamol with ascorbic acid and pheniramine. Ascorbic acid was an important agent in common cold treatment for years. Current data suggest that its effect is small, but might be of statistical importance, although we believe that shortening the cold duration by 1.6 days has low clinical significance [27]. There is no data suggesting its efficacy in adults [52]. One might argue that this set of old generic medication is outdated, while some active substances cause several adverse events, and several new OTC agents sold separately are available on the market [17, 35]. However, this combination is much cheaper than NSAIDs, mucolytic or cough suppressant and alpha-mimetics sold separately (in Poland it may be 1:3 price ratio). Their drawback is that they are sold separately, and not present in combinations (at least in Poland) or in a soluble form [35, 39].

Furthermore, the second most important class of products was nasal decongestant, recommended in one in three patient encounters. It is in line with the papers suggesting common cold treatment [53]. Interestingly, inosines were also advised in one out of three cases. Utilizing this anti-viral agent of unproven efficacy [54], although one small study suggesting otherwise [55], is probably a result of advertising. In almost every third case the pharmacists recommended OTC mucolytics. This class of agents is prescribed in lower respiratory tract infections, chronic bronchitis, pneumonia, in patients with expectorant problems [56]. There are no reasons for utilizing mucoactive agents in upper respiratory viral infections. Their utilization in acute rhinosinusitis does not have reasonable clinical trial evidence [57]. The second generation anti-histamines are widely utilized in common cold treatment, although there are no clinical trials supporting the benefits of this approach [39]. Taken together, clinically relevant symptomatic common cold treatment should base on using analgesics (NSAIDs and paracetamol), decongestant, anti-histamines (but in combination with other classes of medications) [44, 51, 54]. This data indicates that in a number of cases the pharmacists failed to recommended products with proven clinical evidence for the effective anti-common cold treatment.

While the pharmacists claimed that their decisions were solely based on the analysis of patient history and symptoms, in most cases their treatment decisions are limited to advising soluble medication combinations. This approach seems to be based on a “one-size-fits-all” rule and does not seem to rely on patient symptoms. This opinion is supported by the fact that symptom-driven advice suggests that only fever seemed to compel the pharmacists to advise NSAIDs. The interesting scenario involves patients who reported cough as the main common cold symptom. It seems not to have changed the pharmacists’ decision. Moreover, most patients did not receive any cough suppressant available over-the-counter. Dry cough is a reasonable indication to suggest such medication [58, 59]. Finally, a pharmacist has less than 5 min to listen to the patient and make a decision. Therefore, their decisions seem to be influenced by lack of training, lack of time to evaluate the patient, lack of awareness of evidence, and marketing strategy (pharmaceutical drug promotion and financial incentives i.e. fulfilling sale plans and target sale bonuses) [60, 61]. In contrary, mucoactive agents are probably the most frequent medication additions advised by the pharmacists during common cold treatment. They were advised without the analysis of cough characteristics [62].

As we suggested above, pharmacist counselling is the second line (after self-medication) of treating common cold. It would be of interest to introduce more symptom-based approach (such as indicated in *Mináriková* et al. study [6]) to ensure that pharmacists understand the symptoms and the mechanism of common cold and acute rhinosinusitis. Moreover, we believe that some formal or informal education should also be introduced (i.e. focused on cough, its types and treatment). Safety of OTC medications also ought to be emphasized, especially their interaction with other drugs and the influence on driving and operating machinery [63, 64].

The study has several limitations. On the one hand, the involvement of qualified pharmacists in this study, as opposed to conducting an on-line or e-mail or phone survey, allows collecting more reliable data. However, the pharmacist counselling was not recorded in any way (i.e. by audio or audiovisual recording), therefore there is a risk of differences between the collected data and the actual state, which would suggest employing the “mystery shopper” methodology for such type of study. In addition, we are not able to check whether the pharmacists duly completed the data collection forms and whether it was done for all the patients seeking pharmacist advice on anti-common cold OTC drugs who met the criteria for inclusion to the survey. Thirdly, in our study the pharmacy counselling was provided only by the pharmacists, not including pharmacy technicians. This approach limited the collected data to only one group of pharmacy employees. Extending the study to both pharmacists and pharmacy technicians should provide more comprehensive information about pharmacist recommendations on OTC products. In addition, this would allow to compare the most commonly advised classes of medications between both groups. Finally, our study was limited to OTC anti-common cold products commonly used in Poland. Therefore, in the data collection form there was an option “other”, that the pharmacists could select. However, the option did not allow the pharmacist to enter additional data about the type of product, and did not provide any additional information about the variety of the advised products, apart from those mentioned in the list. For this reason, it would be beneficial to improve the methodological approach by extending the data collection form to all commercially available preparations or to allow specific products to be listed in the “other” option.

## Conclusion

Clinically relevant symptomatic common cold treatment is based on using analgesics (NSAIDs and paracetamol), decongestant, anti-histamines (but in combination with other classes of medications) and cough suppressants. According to our data, the most common approach for pharmacist recommendations on anti-common cold treatment was a “shotgun” approach. The pharmacists commonly made recommendations for products that lack strong evidence for efficacy (i.e. anti-viral agents) and are potentially unnecessary based on the symptom presentation. The possible explanation for the pharmacists’ recommendations may include lack of training, lack of time to evaluate the patient, lack of awareness of evidence and recommendations based on pharmaceutical company promotion and financial incentives.

## Supplementary Information

**Additional file 1.** Questionnaire.

**Additional file 2: Suppl Figure 1.** Searching strategy for randomized clinical trials on common cold treatment efficiency (Cochrane Library and PubMed database).

**Additional file 3: Suppl Table 1.** Effective, possibly effective and ineffective symptomatic common cold treatment in adults.

## Data Availability

All data generated or analyzed during this study are included in this published article [and its supplementary information files].

## Abbreviations

OTC: Over-the-counter
NSAIDs: Nonsteroidal anti-inflammatory drugs
Phenylephrine HCL: Phenylephrine hydrochloride
