# Supplementary material for: Pharmacist’s recommendations of over-the-counter treatments for the common cold - analysis of prospective cases in Poland
Source: BMC Fam Pract. 2021 Oct 30;22:216. doi: 10.1186/s12875-021-01561-2 (PMC8556806; doi:10.1186/s12875-021-01561-2)
Supplement: Supplementary file 1 — Additional file 1. Questionnaire. [file 12875_2021_1561_MOESM1_ESM.docx]

**QUESTIONNAIRE**

**AGE**

**□** 18-40 **□** 40-60 **□** 60-90

**SEX**

**□** women **□** men

**SYMPTOMS**

**□** headache **□** fever

**□** rhinorrhoea **□** cough **□** fatigue

**BASIC MEDICATIONS**

**Soluble formulation:**

**□** Apap Anti-Cold **□** Coldrex Max Grip **□** Febrisan

**□** Fervex **□** Gripex Hot **□** Gripex Hot Max

**□** Polopiryna Complex **□** Pyralgina Sprint **□**Theraflu Extra Grip

**□**Theraflu Sinus **□**Vicks **□**other

**Tablets/Capsules:**

**□** Acatar Anti Tabs **□**Aspirin **□** Aspirin C

**□** Aspirin Complex **□** Coldrex Max Grip **□** Eloprine

**□**Engystol **□** Esberitox **□** Gripex

**□** Gripex Max **□** Groprinosin **□**Ibuprom Sinus

**□** Neosine **□** Oscillococcinum **□** Tabcin

**□** others

**ADD-ON MEDICATIONS (different formulations)**

**□** ACC **□** Allegra **□** Allertec

**□** Chlorchinaldin **□** Cholinex **□** Deflegmin

**□** Flavamed **□** Flegamina **□** Loratan

**□** Mucosolvan **□** Neo Angin **□** Otrivin

**□** Strepsils **□** Sudafed Xylo spray **□** Vicks spray

**□** Xylorhin **□** others

**DECISION RATIONALE**

**□**medicament price **□** in-depth patient’s interview

**□** knowledge and belief about medicament effectiveness

**□** other (marketing policy, bonuses for sale achievements, etc.)
