# Supplementary material for: Pharmacist’s recommendations of over-the-counter treatments for the common cold - analysis of prospective cases in Poland
Source: BMC Fam Pract. 2021 Oct 30;22:216. doi: 10.1186/s12875-021-01561-2 (PMC8556806; doi:10.1186/s12875-021-01561-2)
Supplement: Supplementary file 2 — Additional file 2: Suppl Figure 1. Searching strategy for randomized clinical trials on common cold treatment efficiency (Cochrane Library and PubMed database). [file 12875_2021_1561_MOESM2_ESM.docx]

Randomized clinical trials identified in PubMed database

(n = 1)

Reviews of randomized clinical trials on common cold treatment identified in Cochrane Library

(n = 28)

**

**

Total number of reviewed trials

(by titles and abstracts)

(n = 29)

Number of trials excluded from the review (based on the exclusion criteria)

(n = 9)

Number of trials reviewed in detail

(based on the inclusion criteria)

(n = 20)

Number of excluded trials

(n = 3)

Number of Cochrane Reviews and PubMed RCTs analyzed in publication

(n =17)

**Suppl Figure 1. Searching strategy for randomized clinical trials on common cold treatment efficiency (Cochrane Library and PubMed database).**
