# Supplementary material for: Pharmacist’s recommendations of over-the-counter treatments for the common cold - analysis of prospective cases in Poland
Source: BMC Fam Pract. 2021 Oct 30;22:216. doi: 10.1186/s12875-021-01561-2 (PMC8556806; doi:10.1186/s12875-021-01561-2)
Supplement: Supplementary file 3 — Additional file 3: Suppl Table 1. Effective, possibly effective and ineffective symptomatic common cold treatment in adults. [file 12875_2021_1561_MOESM3_ESM.docx]

**Suppl Table 1. Effective, possibly effective and ineffective symptomatic common cold treatment in adults.**

| Review type | Treatment type | Type of study | Number of participants | Effect of treatment | Comment |
| --- | --- | --- | --- | --- | --- |
| Cochrane review  [34] | **acetaminophen (paracetamol)** | 4 RCTs | n=758 | Headache relieving (effective in 2 RCTs; no significant change in 1 RCT) | Inconsistent results |
|  |  |  |  |  | Different data scales for results reporting |
|  |  |  |  | Improving nasal obstruction (effective in 1 RCT; no statistical significance in 1 RCT) |  |
|  |  |  |  | Subjective changes in achiness (effective in 1 RCT; no correlation in 3 RCTs) | Intervention group doses vary between studies (between 500mg - 1000 mg every six hours) |
|  |  |  |  | Sore throat (no significant effect) |  |
|  |  |  |  | Adverse events - mild to moderate (exception 2 cases of severe nausea) |  |
| Cochrane review  [39] | **anti-histamines (monotherapy)** | 18 RCTs | n=4342  (including 212 children) | For adults: beneficial effect of antihistamine monotherapy on symptom severity, 45% vs 38% (intervention vs control), OR=0.74, 95%CI 0.6-0.92 (for short-term usage) | Beneficial effect for short-term usage (1-2 days), no effects for mid- and long-term usage |
|  |  |  |  | No significant effect for mild-term usage (up to 304 days) or long-term usage (up to 6-10 days) | Effects on separate cold symptoms are not clinically significant |
|  |  |  |  | Beneficial effect on rhinorrhea (day 3), MD=-0.23, 95%CI -0.39 -0.06 (four-five point severity scale), beneficial effect on sneezing (day 3), MD=-0.35, 95%CI -0.49 -0.20 (four point severity scale) |  |
|  |  |  |  | Adverse events: sedation (differences not statistically significant) |  |
| Cochrane review  [38] | **ipratropium bromide (intranasal)** | 7 RCTs | n=2144 | 4 RCTs, n=1959 participants - subjective change in rhinorrhea severity; for 4 RCTs no significant difference between groups for nasal congestion; for 2 RCTs positive response on intervention | Limitations of current evidence |
|  |  |  |  | Adverse events: nasal dryness, epistaxis, blood tinged mucus; OR=2.09, 95%CI 1.40-3.11 | Possibly effective on rhinorrhea, no effect on nasal congestion and possible side effects (greater than placebo) |
| Cochrane review  [35] | **NSAIDs** | 9 RCTs | n=1069 | No significant reduction in the total symptom score, SMD=-0.40, 95%CI -1.03- 0.24 | Diversity of study results |
|  |  |  |  | No significant reduction in cold duration, MD=-0.23, 95%CI -1.75-1.29 | Clear analgesic effect but no clear evidence in improvement of respiratory symptoms |
|  |  |  |  | No significant improvement for cough score, SMD=-0.05, 95%CI -0.66-0.56 |  |
|  |  |  |  | Significant improve for sneezing score, SMD=-0.44, 95%CI -0.75 -0.12 |  |
|  |  |  |  | Significant benefits for using NSAIDs as analgesics |  |
|  |  |  |  | Adverse events: risk of AE not high, RR=2.94, 95%CI 0.51-17.03 |  |
| Cochrane review  [36] | **nasal decongestants (monotherapy)** | 15 RCTs | n=1838 | Single-dose decongestant: 10 RCTs - treatment effectiveness tested after 15min-10 hours - huge diversity of the results reporting; results based on the subjective symptom scores (for 7/10 RCTs); | Only limited evidence for single-dose of decongestant - hard to determined clear conclusions about effectiveness |
|  |  |  | 14 RCTs on adults | Multi-dose decongestant: 9 RCTs - treatment effectiveness tested after 3 hours after last dose - small clinical effect - SMD=0.49, 95%CI 0.07-0.92, p=0.002 | Only small positive effect for multi-dose decongestant treatment of common cold |
|  |  |  |  | Adverse events: for single-dose decongestant - in 2 RCTs reported but without statistically significance; for multi-dose decongestant: AE 126/1000 vs 125/1000 (intervention vs control), OR=0.98, 95%CI 0.68-1.40, p=0.9 | Insufficient good quality evidence for concluding about effectiveness of multi-dose of decongestant in common cold |
| Cochrane review  [16] | **antihistamine-decongestant-analgesic (combination)** | 27 RCTs | n=5117 | Antihistamine-decongestant (14 RCTs) - for 6 RCTS, n=621 participants - treatment failure OR=0.27, 95% CI 0.15-0.50; NNTB=4 (95% CI 3-5.6); favorable response to treatment - 66% vs 41% (intervention vs control); other trials shown global effectiveness data | Antihistamine-analgesic-decongestant - some beneficial effect in adults, however increased risk of different adverse events |
|  |  |  |  | Antihistamine-analgesic (3 RCTs) - for one RCT, n=582 participants - treatment failure OR=0.33, 95% CI 0.23-0.46; NNTB=6.67, 95% CI 4.76-12.5; 6-days treatment - cure of 70% vs 43% (intervention vs control - ascorbic acid); two additional reported global effectiveness data | Antihistamine-decongestant (2 RCTs), n=113 children - any significant effect of intervention |
|  |  |  |  | Analgesic-decongestant (6 RCTs) - benefits from intervention - 73% vs 52% (intervention vs control-paracetamol), OR=0.28, 95% cI 0.15-0.52; one trial reported global effectiveness |  |
|  |  |  |  | Antihistamine-analgesic-decongestant (5 RCTs) - four reported global effectiveness data 52% vs 34% (intervention vs control), treatment failure OR=0.47, 95% CI 0.33-0.67; NNTB=5.6, 95% CI 3.8-10.2; for 2 RCTs - no effect; for 2 RCTs - no benefits |  |
|  |  |  |  | Adverse events: for antihistamine-decongestant - 19% vs 13% (intervention vs treatment) OR=1.58, 95% CI 0.78-3.21; for analgesic-decongestant - OR=1.71, 95% CI 1.23-3.21, NNTH=14; for antihistamine-analgesic - 12% vs 10% (intervention vs control), OR=1.27, 95% CI 0.50-3.23; for antihistamine-analgesic-decongestant - 2% vs 4% (intervention vs control in one RCT, for second RCT - no differences between groups |  |
| Systematic review (PubMed) [37] | **xylometazoline (alone) and in combination with ipratropium bromide** | 4 RCTs | no data of number of participants | For monotherapy - clinically relevant decongestant effect (up to 10 hours); nasal conductance 69.7% greater than control (in 1 hour, for 10 hours p=0.0009), p=0.001; peak subjective effect - 20.7 vs 31.5 (intervention vs control), p=0,0298; total common cold symptom score - 25.71 vs 35.79 (intervention vs control, p=0,0221 (for 1 day) | Xylometazoline in monotherapy effective against nasal congestion |
|  |  |  |  | For combination with ipratropium - effectiveness against nasal congestion and rhinorrhea (up to 7 days), p<0.05 | Xylometazoline in combination with ipratropium bromide effective against nasal congestion and rhinorrhea |
|  |  |  |  | Adverse events: mild to moderate - headache (3.4% patients), period pain 10.3% patients), epistaxis (3.4% patients), blood-tinged mucus (10-26% patients) |  |
| Cochrane review  [40] | **nasal saline irrigation** | 5 RCTs | n=749 (enrolled) n=565 (data providing) | For 2 RCTs, n=11 patients: days to wellness - 1.11-2.58 vs 9.24 (intervention vs control) | Limited evidence that treatment is effective, further larger scale trials needed |
|  |  |  |  | For 2 RCTs, n=422 patients: antibiotic usage - 60 per 1000 vs 89 per 100, OR=0.65, 95%CI 0.29-1.46 | 1 RCT n=401 children (aged 6-10) significant reduction of number of symptoms: nasal secretion, sore throat, nasal breathing score, nasal obstruction, reduction in usage of additional nasal decongestant |
|  |  |  |  | For 1 RCTs, n=390 patients: sore throat score: 0.004-0.24 vs 1.23 points (intervention vs control) | Possible benefits for relieving symptoms of acute upper respiratory tract infection |
| Cochrane review  [42] | **corticosteroids (intranasal)** | 3 RCTs | n=353 | 1 RCT, n=53 participants, mean number of symptomatic days - 10.7 vs 10.3 (intervention vs control), p=0,72; mean time to recovery - 12 days vs 11 days (intervention vs control), p=0.81 | 1 RCT, n=100 children (between 2-14 years) - inadequate reporting of symptom score measurement |
|  |  |  |  | 1 RCT, n=199 participants, no significant difference on symptom duration between groups; mean duration of sore throat - 5.3 days vs 3.7 days (intervention vs control), p<0.001 | No clear evidence on the beneficial effect of corticosteroid usage in common cold |
|  |  |  |  | Adverse events: secondary bacterial infection, no statistically significant difference between groups |  |
| Cochrane review  [27] | **vitamin C** | 29 RCTs on efficiency of vit C on common cold prevention | n=11306 | General for trials, n=10708 participants - RR=0.97, 95%CI 0.94-1.00 | Vitamin C supplementation may be useful during brief periods of physical exercises |
|  |  | 31 RCTs on effect of vit C on common cold duration |  | For 5 RCTs, n=598 - RR=0.48, 95% CI 0.35-0.64 | No confirmed results on vitamin C efficiency in therapeutic trials |
|  |  |  |  | Reduction of colds duration: 3-12% (adults), 7-21% children |  |
|  |  |  |  | Any consistent effect of vitamin C on common cold (duration of severity) in therapeutic trials |  |
| Cochrane review  [28] | **zinc** | 18 RCTs | n=1387 in 16 therapeutic RCTs | Significant reduction of common cold symptom duration, MD=-1.03, 95%CI -1.72 -0.34, p=0.003; No effect on symptom severity, MD=-1.06, 95%CI -2.36-0.23, p=0.11; smaller number of symptomatic patients after 7 days zinc intake (intervention vs control), OR=0.45, 95%CI 0.2-1.0, p=0.05; cold developing IRR=0.64, 95%CI 0.47-0.88, p=0.006 | Large clinical trials needed due to heterogeneity of the data |
|  |  |  | n=394 in 2 preventive RCTs | Adverse events: bad taste, OR=2.31, 95%CI 1.71-3.11, p<0.001 nausea, OR=2.15, 95%CI 1.44-3.23, p=0.002 | Reduction of common cold symptoms where administered up to 24 hours after first symptoms |
|  |  |  |  |  | More prophylactic supplementation than treatment, especially with AE occurrence |
| Cochrane review  [26] | **garlic** | 1 RCT | n=146 | 24 vs 65 common cold event (intervention vs placebo) p<0.001 | Insufficient clinical trial evidence |
|  |  |  |  | length of illness similar 4.63 vs 5.63 days (intervention vs placebo) | Only single trial with self-reported episodes of common cold |
|  |  |  |  | Adverse event - rash and odor | 3 months (garlic vs placebo) more preventive than treatment |
|  |  |  |  |  | Large double-blind RCT should be provided to provide conclusive evidence |
| Cochrane review  [29] | **echinacea** | 24 RCTs | n=4631 | Small preventive effect | Variety of tested products and results make it impossible to draw clear conclusions |
|  |  |  |  | No statistical significance for disease occurrence reduction | There is possible weak beneficial effect but with questionable clinical relevance |
| Cochrane review  [31] | ***Pelargonium sidoides* extract** | 8 RCTs | n=746 in 3 RCTs for acute bronchitis | For trials of efficiency in acute bronchitis - effectiveness for liquid preparation - RR=0.66, 95% CI 0.52-0.83 (no significant results for tablets - RR=0.95, 95% CI 0.91-0.99) | Statistical heterogeneity, low quality of evidence |
|  |  |  | n=103 in 1 RCT for sinusitis | For trials of efficiency on sinusitis - RR=0.43, 95% CI 0.30-0.62; complete resolution ad day 21 | Including 3 RCTs on children (n=819) - effectiveness for liquid preparation for acute bronchitis RR=0.82, 95% CI 0.77-0.88 (for tablets RR=0.96, 95% CI 0.89-1.03) |
|  |  |  | n=103 in 1 RCT for efficiency in common cold | For trial on efficiency on common cold - statistical significance after 10 days (no effect after 5 days - RR=0.96, 95%CI 0.9-1.09) |  |
|  |  |  |  | Adverse events: nausea, diarrhea, vomiting, allergic skin reaction, |  |
| Cochrane review  [30] | **Chinese medicine herbs** | 17 RCTs | n=3212 | Risk of bias was very high to make any conclusion about effect of herbal products on common cold | Evidence did not support recommendation of Chinese medicine herbs for common cold |
|  |  |  |  |  | Another clinical trials are required |
| Cochrane review  [43] | **anti-viral agent** | 241 RCTs | N/A | Evaluated protective effect on experimental or natural colds of different anti-viral agents, i.e. interferons, dipyridamole, palmitate | No clear evidence on effectiveness of antiviral usage on common cold treatment |
|  |  |  |  |  | None licensed agent for common cold treatment |
|  |  |  |  | For prolonged usage increased incidences of blood-tinged nasal discharge (for interferons), OR=4.52, 95%CI 3.78-5.41 |  |
| Cochrane review  [41] | **antibiotics** | 11 RCTs | n=1047 | No significant difference between intervention and control for symptoms persistence or lack of cure, RR=0.95. 95%CI 0.59-1.51 | n=791 participants (5 RCTs on rhinitis) |
|  |  |  | 6 RCTs on common cold | Adverse events: RR=1.8, 95%CI 1.01-3.21; RR of AR for adults: 2.62, 95%CI 1.32-5.18; RR of AR for children: RR=0.91, 95%CI 0.51-1.63 | No evidence on the effectiveness of antibiotics on common cold |
| Review type | Treatment type | Type of study | Number of participants | Effect of treatment | Comment |
| Cochrane review  [32] | **acetaminophen (paracetamol)** | 4 RCTs | n=758 | Headache relieving (effective in 2 RCTs; no significant change in 1 RCT) | Inconsistent results |
|  |  |  |  |  | Different data scales for results reporting |
|  |  |  |  | Improving nasal obstruction (effective in 1 RCT; no statistical significance in 1 RCT) |  |
|  |  |  |  | Subjective changes in achiness (effective in 1 RCT; no correlation in 3 RCTs) | Intervention group doses vary between studies (between 500mg - 1000 mg every six hours) |
|  |  |  |  | Sore throat (no significant effect) |  |
|  |  |  |  | Adverse events - mild to moderate (exception 2 cases of severe nausea) |  |
| Cochrane review  [37] | **anti-histamines (monotherapy)** | 18 RCTs | n=4342  (including 212 children) | For adults: beneficial effect of antihistamine monotherapy on symptom severity, 45% vs 38% (intervention vs control), OR=0.74, 95%CI 0.6-0.92 (for short-term usage) | Beneficial effect for short-term usage (1-2 days), no effects for mid- and long-term usage |
|  |  |  |  | No significant effect for mild-term usage (up to 304 days) or long-term usage (up to 6-10 days) | Effects on separate cold symptoms are not clinically significant |
|  |  |  |  | Beneficial effect on rhinorrhea (day 3), MD=-0.23, 95%CI -0.39 -0.06 (four-five point severity scale), beneficial effect on sneezing (day 3), MD=-0.35, 95%CI -0.49 -0.20 (four point severity scale) |  |
|  |  |  |  | Adverse events: sedation (differences not statistically significant) |  |
| Cochrane review  [36] | **ipratropium bromide (intranasal)** | 7 RCTs | n=2144 | 4 RCTs, n=1959 participants - subjective change in rhinorrhea severity; for 4 RCTs no significant difference between groups for nasal congestion; for 2 RCTs positive response on intervention | Limitations of current evidence |
|  |  |  |  | Adverse events: nasal dryness, epistaxis, blood tinged mucus; OR=2.09, 95%CI 1.40-3.11 | Possibly effective on rhinorrhea, no effect on nasal congestion and possible side effects (greater than placebo) |
| Cochrane review  [33] | **NSAIDs** | 9 RCTs | n=1069 | No significant reduction in the total symptom score, SMD=-0.40, 95%CI -1.03- 0.24 | Diversity of study results |
|  |  |  |  | No significant reduction in cold duration, MD=-0.23, 95%CI -1.75-1.29 | Clear analgesic effect but no clear evidence in improvement of respiratory symptoms |
|  |  |  |  | No significant improvement for cough score, SMD=-0.05, 95%CI -0.66-0.56 |  |
|  |  |  |  | Significant improve for sneezing score, SMD=-0.44, 95%CI -0.75 -0.12 |  |
|  |  |  |  | Significant benefits for using NSAIDs as analgesics |  |
|  |  |  |  | Adverse events: risk of AE not high, RR=2.94, 95%CI 0.51-17.03 |  |
| Cochrane review  [34] | **nasal decongestants (monotherapy)** | 15 RCTs | n=1838 | Single-dose decongestant: 10 RCTs - treatment effectiveness tested after 15min-10 hours - huge diversity of the results reporting; results based on the subjective symptom scores (for 7/10 RCTs); | Only limited evidence for single-dose of decongestant - hard to determined clear conclusions about effectiveness |
|  |  |  | 14 RCTs on adults | Multi-dose decongestant: 9 RCTs - treatment effectiveness tested after 3 hours after last dose - small clinical effect - SMD=0.49, 95%CI 0.07-0.92, p=0.002 | Only small positive effect for multi-dose decongestant treatment of common cold |
|  |  |  |  | Adverse events: for single-dose decongestant - in 2 RCTs reported but without statistically significance; for multi-dose decongestant: AE 126/1000 vs 125/1000 (intervention vs control), OR=0.98, 95%CI 0.68-1.40, p=0.9 | Insufficient good quality evidence for concluding about effectiveness of multi-dose of decongestant in common cold |
| Cochrane review  [16] | **antihistamine-decongestant-analgesic (combination)** | 27 RCTs | n=5117 | Antihistamine-decongestant (14 RCTs) - for 6 RCTS, n=621 participants - treatment failure OR=0.27, 95% CI 0.15-0.50; NNTB=4 (95% CI 3-5.6); favorable response to treatment - 66% vs 41% (intervention vs control); other trials shown global effectiveness data | Antihistamine-analgesic-decongestant - some beneficial effect in adults, however increased risk of different adverse events |
|  |  |  |  | Antihistamine-analgesic (3 RCTs) - for one RCT, n=582 participants - treatment failure OR=0.33, 95% CI 0.23-0.46; NNTB=6.67, 95% CI 4.76-12.5; 6-days treatment - cure of 70% vs 43% (intervention vs control - ascorbic acid); two additional reported global effectiveness data | Antihistamine-decongestant (2 RCTs), n=113 children - any significant effect of intervention |
|  |  |  |  | Analgesic-decongestant (6 RCTs) - benefits from intervention - 73% vs 52% (intervention vs control-paracetamol), OR=0.28, 95% cI 0.15-0.52; one trial reported global effectiveness |  |
|  |  |  |  | Antihistamine-analgesic-decongestant (5 RCTs) - four reported global effectiveness data 52% vs 34% (intervention vs control), treatment failure OR=0.47, 95% CI 0.33-0.67; NNTB=5.6, 95% CI 3.8-10.2; for 2 RCTs - no effect; for 2 RCTs - no benefits |  |
|  |  |  |  | Adverse events: for antihistamine-decongestant - 19% vs 13% (intervention vs treatment) OR=1.58, 95% CI 0.78-3.21; for analgesic-decongestant - OR=1.71, 95% CI 1.23-3.21, NNTH=14; for antihistamine-analgesic - 12% vs 10% (intervention vs control), OR=1.27, 95% CI 0.50-3.23; for antihistamine-analgesic-decongestant - 2% vs 4% (intervention vs control in one RCT, for second RCT - no differences between groups |  |
| Systematic review (PubMed) [35] | **xylometazoline (alone) and in combination with ipratropium bromide** | 4 RCTs | no data of number of participants | For monotherapy - clinically relevant decongestant effect (up to 10 hours); nasal conductance 69.7% greater than control (in 1 hour, for 10 hours p=0.0009), p=0.001; peak subjective effect - 20.7 vs 31.5 (intervention vs control), p=0,0298; total common cold symptom score - 25.71 vs 35.79 (intervention vs control, p=0,0221 (for 1 day) | Xylometazoline in monotherapy effective against nasal congestion |
|  |  |  |  | For combination with ipratropium - effectiveness against nasal congestion and rhinorrhea (up to 7 days), p<0.05 | Xylometazoline in combination with ipratropium bromide effective against nasal congestion and rhinorrhea |
|  |  |  |  | Adverse events: mild to moderate - headache (3.4% patients), period pain 10.3% patients), epistaxis (3.4% patients), blood-tinged mucus (10-26% patients) |  |
| Cochrane review  [38] | **nasal saline irrigation** | 5 RCTs | n=749 (enrolled) n=565 (data providing) | For 2 RCTs, n=11 patients: days to wellness - 1.11-2.58 vs 9.24 (intervention vs control) | Limited evidence that treatment is effective, further larger scale trials needed |
|  |  |  |  | For 2 RCTs, n=422 patients: antibiotic usage - 60 per 1000 vs 89 per 100, OR=0.65, 95%CI 0.29-1.46 | 1 RCT n=401 children (aged 6-10) significant reduction of number of symptoms: nasal secretion, sore throat, nasal breathing score, nasal obstruction, reduction in usage of additional nasal decongestant |
|  |  |  |  | For 1 RCTs, n=390 patients: sore throat score: 0.004-0.24 vs 1.23 points (intervention vs control) | Possible benefits for relieving symptoms of acute upper respiratory tract infection |
| Cochrane review  [40] | **corticosteroids (intranasal)** | 3 RCTs | n=353 | 1 RCT, n=53 participants, mean number of symptomatic days - 10.7 vs 10.3 (intervention vs control), p=0,72; mean time to recovery - 12 days vs 11 days (intervention vs control), p=0.81 | 1 RCT, n=100 children (between 2-14 years) - inadequate reporting of symptom score measurement |
|  |  |  |  | 1 RCT, n=199 participants, no significant difference on symptom duration between groups; mean duration of sore throat - 5.3 days vs 3.7 days (intervention vs control), p<0.001 | No clear evidence on the beneficial effect of corticosteroid usage in common cold |
|  |  |  |  | Adverse events: secondary bacterial infection, no statistically significant difference between groups |  |
| Cochrane review  [27] | **vitamin C** | 29 RCTs on efficiency of vit C on common cold prevention | n=11306 | General for trials, n=10708 participants - RR=0.97, 95%CI 0.94-1.00 | Vitamin C supplementation may be useful during brief periods of physical exercises |
|  |  | 31 RCTs on effect of vit C on common cold duration |  | For 5 RCTs, n=598 - RR=0.48, 95% CI 0.35-0.64 | No confirmed results on vitamin C efficiency in therapeutic trials |
|  |  |  |  | Reduction of colds duration: 3-12% (adults), 7-21% children |  |
|  |  |  |  | Any consistent effect of vitamin C on common cold (duration of severity) in therapeutic trials |  |
| Cochrane review  [28] | **zinc** | 18 RCTs | n=1387 in 16 therapeutic RCTs | Significant reduction of common cold symptom duration, MD=-1.03, 95%CI -1.72 -0.34, p=0.003; No effect on symptom severity, MD=-1.06, 95%CI -2.36-0.23, p=0.11; smaller number of symptomatic patients after 7 days zinc intake (intervention vs control), OR=0.45, 95%CI 0.2-1.0, p=0.05; cold developing IRR=0.64, 95%CI 0.47-0.88, p=0.006 | Large clinical trials needed due to heterogeneity of the data |
|  |  |  | n=394 in 2 preventive RCTs | Adverse events: bad taste, OR=2.31, 95%CI 1.71-3.11, p<0.001 nausea, OR=2.15, 95%CI 1.44-3.23, p=0.002 | Reduction of common cold symptoms where administered up to 24 hours after first symptoms |
|  |  |  |  |  | More prophylactic supplementation than treatment, especially with AE occurrence |
| Cochrane review  [26] | **garlic** | 1 RCT | n=146 | 24 vs 65 common cold event (intervention vs placebo) p<0.001 | Insufficient clinical trial evidence |
|  |  |  |  | length of illness similar 4.63 vs 5.63 days (intervention vs placebo) | Only single trial with self-reported episodes of common cold |
|  |  |  |  | Adverse event - rash and odor | 3 months (garlic vs placebo) more preventive than treatment |
|  |  |  |  |  | Large double-blind RCT should be provided to provide conclusive evidence |
| Cochrane review  [29] | **echinacea** | 24 RCTs | n=4631 | Small preventive effect | Variety of tested products and results make it impossible to draw clear conclusions |
|  |  |  |  | No statistical significance for disease occurrence reduction | There is possible weak beneficial effect but with questionable clinical relevance |
| Cochrane review  [31] | ***Pelargonium sidoides* extract** | 8 RCTs | n=746 in 3 RCTs for acute bronchitis | For trials of efficiency in acute bronchitis - effectiveness for liquid preparation - RR=0.66, 95% CI 0.52-0.83 (no significant results for tablets - RR=0.95, 95% CI 0.91-0.99) | Statistical heterogeneity, low quality of evidence |
|  |  |  | n=103 in 1 RCT for sinusitis | For trials of efficiency on sinusitis - RR=0.43, 95% CI 0.30-0.62; complete resolution ad day 21 | Including 3 RCTs on children (n=819) - effectiveness for liquid preparation for acute bronchitis RR=0.82, 95% CI 0.77-0.88 (for tablets RR=0.96, 95% CI 0.89-1.03) |
|  |  |  | n=103 in 1 RCT for efficiency in common cold | For trial on efficiency on common cold - statistical significance after 10 days (no effect after 5 days - RR=0.96, 95%CI 0.9-1.09) |  |
|  |  |  |  | Adverse events: nausea, diarrhea, vomiting, allergic skin reaction, |  |
| Cochrane review  [30] | **Chinese medicine herbs** | 17 RCTs | n=3212 | Risk of bias was very high to make any conclusion about effect of herbal products on common cold | Evidence did not support recommendation of Chinese medicine herbs for common cold |
|  |  |  |  |  | Another clinical trials are required |
| Cochrane review  [41] | **anti-viral agent** | 241 RCTs | N/A | Evaluated protective effect on experimental or natural colds of different anti-viral agents, i.e. interferons, dipyridamole, palmitate | No clear evidence on effectiveness of antiviral usage on common cold treatment |
|  |  |  |  |  | None licensed agent for common cold treatment |
|  |  |  |  | For prolonged usage increased incidences of blood-tinged nasal discharge (for interferons), OR=4.52, 95%CI 3.78-5.41 |  |
| Cochrane review  [39] | **antibiotics** | 11 RCTs | n=1047 | No significant difference between intervention and control for symptoms persistence or lack of cure, RR=0.95. 95%CI 0.59-1.51 | n=791 participants (5 RCTs on rhinitis) |
|  |  |  | 6 RCTs on common cold | Adverse events: RR=1.8, 95%CI 1.01-3.21; RR of AR for adults: 2.62, 95%CI 1.32-5.18; RR of AR for children: RR=0.91, 95%CI 0.51-1.63 | No evidence on the effectiveness of antibiotics on common cold |

Abbreviations: AR – adverse event; CI – confidence interval; IRR – incidence rate ratio; MD – mean difference; NNTB – number needed to treat for an additional beneficial outcome; NNTH – number needed to treat for an additional harmful outcome; NSAIDs – non-steroidal anti-inflammatory drugs; OR- odds ratio; RCT – randomized clinical trials; RR - risk ratio; SMD – standardized mean difference
